# Raised-Volume Forced Expiratory Flow-Volume Curve in Healthy Taiwanese Infants

**DOI:** 10.1038/s41598-017-06815-7

**Published:** 2017-07-24

**Authors:** Shen-Hao Lai, Sui-Ling Liao, Tsung-Chieh Yao, Ming-Han Tsai, Man-Chin Hua, Chih-Yung Chiu, Kuo-Wei Yeh, Jing-Long Huang

**Affiliations:** 1 Department of Pediatrics, Chang Gung Memorial Hospital, Taoyuan, Taiwan; 2 Department of Pediatrics, Chang Gung University, Taoyuan, Taiwan; 3 Department of Pediatrics, Chang Gung Memorial Hospital, Keelung, Taiwan; 4 Prediction of Allergies in Taiwanese Children (PATCH) cohort study, Keelung, Taiwan

## Abstract

The raised-volume rapid thoracoabdominal compression (RVRTC) manoeuvre has been applied to obtain full forced expiratory flow-volume curves in infants. No reference data are available for Asian populations. This study was conducted to establish predictive reference equations for Taiwanese infants. Full-term infants without any chronic disease or major anomaly were enrolled from this cohort study. Full forced expiratory flow-volume curves were acquired using RVRTC manoeuvres through Jaeger’s system. Tidal breath analysis, passive respiratory mechanics, and tidal forced expiratory flow-volume curves were performed and collected at the same measurement. Multiple linear analyses were used to model the variables. We performed 117 tests of RVRTC flow-volume curves in 97 infants. The results revealed that all parameters, except for FEV_0.5_ /FVC, correlated highly and positively with body length. These parameters correlated significantly with other parameters of passive respiratory mechanics and tidal forced expiratory flow-volume curves. This is the first study to establish equipment-specific reference data of full forced expiration using RVRTC manoeuvres in Asian infants. The results revealed that parameters of RVRTC manoeuvres are moderately related to other parameters of infant lung function. These race-specific reference data can be used to more precisely and efficiently diagnose respiratory diseases in infants of Chinese ethnicity.

## Introduction

Many chronic paediatric respiratory diseases, such as bronchopulmonary dysplasia, asthma, and cystic fibrosis, originate early in life. However, techniques available for investigating the respiratory function of young children, particularly infants, are limited. Infant lung function (ILF) testing has been shown to be useful for the early diagnosis of and efficient intervention in lung diseases^1–4^. In the past, a partial expiratory flow-volume curve obtained using the rapid thoracoabdominal compression (RTC) manoeuvre has been used to assess airway function^5–7^. However, because this curve is collected only in the range of tidal volume, flow limitation is occasionally difficult to achieve in healthy infants^8, 9^. In addition, the maximal expiratory flow measured in functional residual capacity (Vmax_FRC_) is relatively unstable because the end-expiratory level may be dynamically high in young infants.

Recently, the raised-volume RTC (RVRTC) technique, initiated near total lung capacity, has been utilised to obtain a full forced expiratory flow-volume curve. This manoeuvre has been shown to differentiate between healthy infants and those with respiratory diseases^5, 10, 11^. During the past 10 years, increasing RVRTC reference data from healthy infants have been reported^8, 12^. The American Thoracic Society/European Respiratory Society (ATS/ERS) task force has developed clinical practice guidelines for the RVRTC technique^5, 11, 13^. However, all the data have been obtained for Caucasian populations and thus studies on individuals of Asian ethnicity are lacking.

The present study was therefore conducted to establish prediction equations for RVRTC data in Taiwanese children during the first 2 years of life. Moreover, after considering sex, age, body weight, and body height, we developed equations that can be expressed as Z-scores. In addition, we investigated the correlation between RVRTC data and other ILF parameters that have been widely used in the past.

## Results

We performed 117 tests in 97 infants (56 boys). Of these, 20 infants received repeated tests at least 5 months apart. The median age, body length, and body weight of the infants were 8 (range, 5–26) months, 78 (range, 63–91) cm, and 10.2 (range, 5.8–15) kg, respectively. All but five infants (who were born at 36 weeks of gestation) were born at ≥37 weeks of gestation. Table 1 summarises the demographic characteristics of the infants.

**Table 1:** Demographic characteristics of 97 infants who underwent 117 lung function tests.

	n*	Mean (SD)	Range
Male, n (%)	97	56 (58%)	
Gestational age, weeks	97	38.4 (1.2)	36–41
Birth weight, kg	97	3.2 (0.4)	2.0–4.4
Birth weight, Z-score	97	−0.1 (0.8)	−2.3–2.4
Birth length, cm	97	50.2 (2.7)	33.0–56.0
Birth length, Z-score	97	−0.1 (0.8)	−2.3–2.4
Weight, Z-score^#^	117	−0.2 (1.0)	−2.6–2.0
Length, Z-score^#^	117	−0.1 (1.2)	−3.6–2.8
Maternal smoking during pregnancy, n (%)	97	3 (3.1%)	
Household smoking, n (%)	97	45 (46.4%)	

Data are expressed as the frequency (%) or mean (SD), unless otherwise stated.

*n describes the number of participants or test occasions.

^#^Body weight and length were obtained during testing.

**Figure 1:**
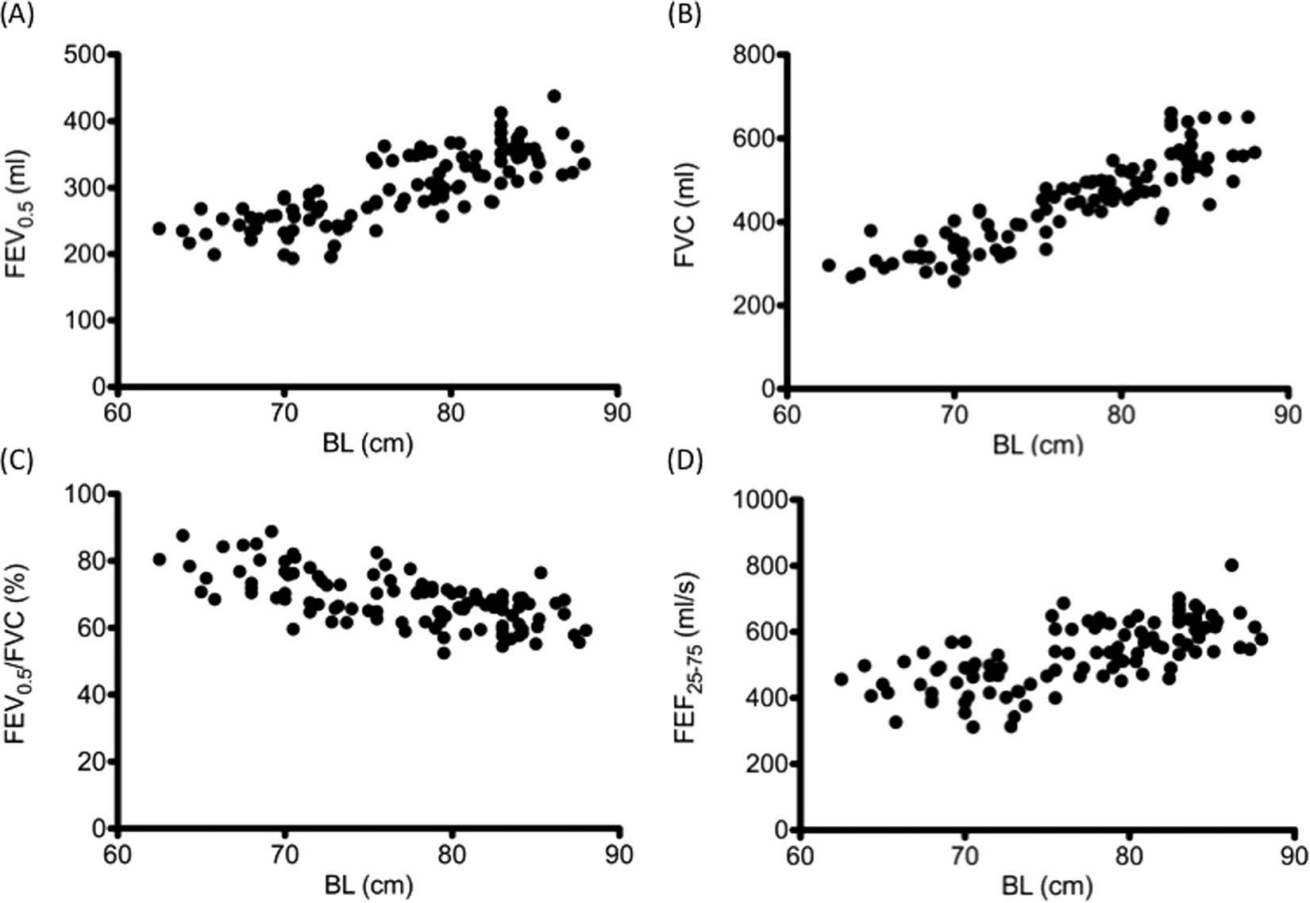
Scatter plots of various raised-volume rapid thoracoabdominal compression parameters. Forced expiratory volume (FEV) at 0.5 sec (FEV_0.5_) (**A**), forced vital capacity (FVC) (**B**), FEV_0.5_/FVC (**C**), and forced midexpiratory flow (FEF_25–75_) (**D**) plotted against body length (BL) at the time of test.

In the stepwise multiple linear regression analysis (variables included were age, body length, body weight, and sex), body length was the most significant independent variable associated with FVC, FEV_1_, FEV_0.75_, FEV_0.5_, FEV_0.4_, FEV_0.3_, FEF_75_, FEF_50_, FEF_25-75_, and PEF. No sex-related differences were observed in the slopes or intercepts of the regression curves. Scatter plots are depicted in Fig. 1. Table 2 lists regression equations for the various parameters of the RVRTC flow-volume curve. All parameters correlated highly with body length. Except the regression curve of FEV_0.5_/FVC, all these parameters exhibited a positive linear correlation with body length. The optimal powers of the regression equation relating body length to FVC, FEV_1_, FEV_0.75_, FEV_0.5_, FEV_0.4_, FEV_0.3_, FEF_75_, FEF_50_, FEF_25–75_, FEV_0.5_/FVC, and PEF were 88%, 86%, 86%, 80%, 76%, 64%, 73%, 69%, 69%, 60%, and 53% respectively. In Table 2, the standard deviations of the residuals were used to compute the Z-scores of any observed values.

**Table 2:** Regression equations of various parameters of infant lung function.

Variable^#^		Coefficient	SE of coefficient*	RSD	R^2^
FVC, ml	Constant (α)	−615.106	54.8	48.079	0.772
	BL, cm (β)	13.752	0.705		
FEV_0.4_, ml	Constant (α)	−137.935	32.253	29.394	0.573
	BL, cm (β)	5.069	0.415		
FEV_0.5_, ml	Constant (α)	−205.169	36.739	32.3	0.634
	BL, cm (β)	6.552	0.473		
FEV_0.75_, ml	Constant (α)	−390.548	43.936	39.773	0.740
	BL, cm (β)	10.02	0.566		
FEV_1_, ml	Constant (α)	−606.480	59.08	53.843	0.737
	BL, cm (β)	13.407	0.76		
FEF_25–75_, ml	Constant (α)	−266.101	79.402	70.95	0.479
	BL, cm (β)	10.325	1.022		
FEF_75_, ml/s	Constant (α)	−327.062	62.396	56.865	0.536
	BL, cm (β)	9.099	0.803		
FEF_50_, ml/s	Constant (α)	−243.795	79.988	72.898	0.473
	BL, cm (β)	10.279	1.029		
FEV_0.5_/FVC, %	Constant (α)	121.233	6.791	6.189	0.355
	BL, cm (β)	−0.683	0.087		
PEF, ml/s	Constant (α)	601.466	22.621	95.911	0.345
	Age, mo (β)	10.912	1.426		

*All P < 0.001.

^#^Function: variables = α + β × BL (or Age).

**Figure 2:**
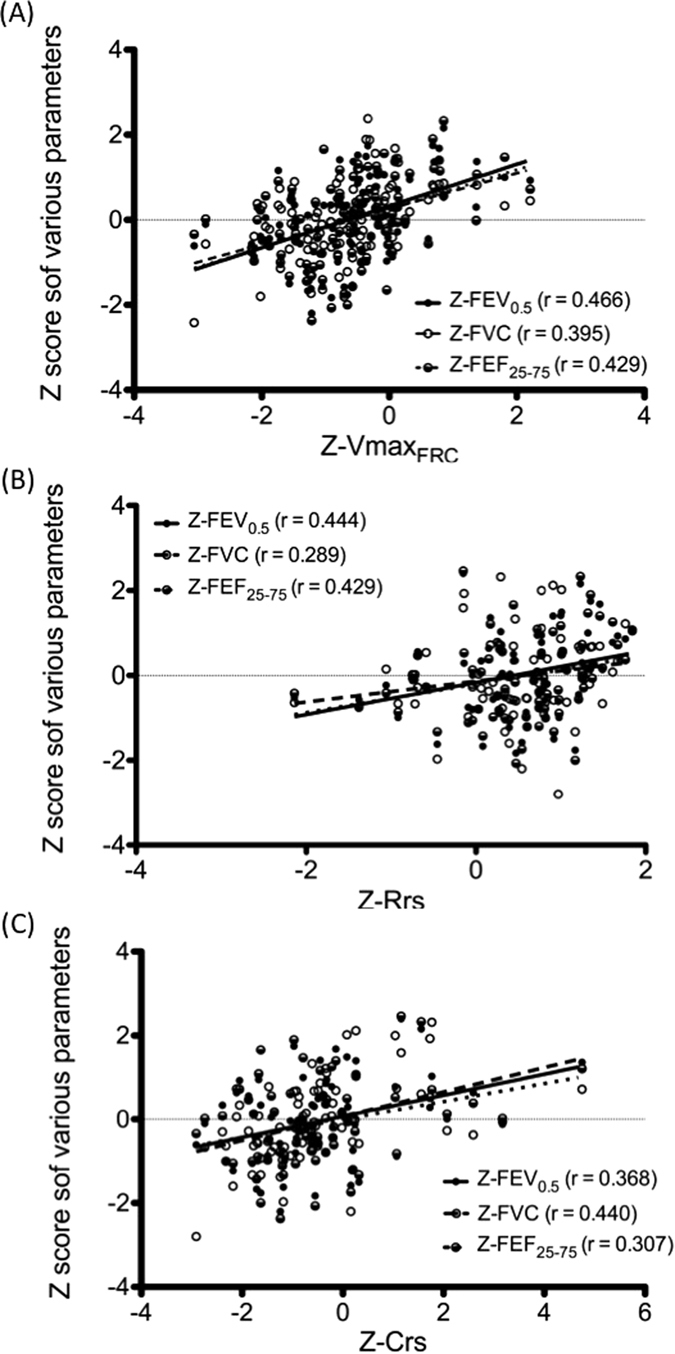
Scatter plots and regression curves of Z-scores of infant lung function parameters. Z-scores of the maximal expiratory flow measured in functional residual capacity (Vmax_FRC_) (**A**), resistance of the respiratory system (Rrs) (**B**), and compliance of the respiratory system (Crs) plotted against the Z-scores of various RVRTC parameters including forced expiratory volume at 0.5 (FEV_0.5_), forced vital capacity (FVC), and forced midexpiratory flow (FEF_25–75_).

To express the measured value as a percentage of that predicated and the between-subject variability into a single number, results of lung function should ideally be mathematically transformed to the demonstration of Z-scores^14^. Thus, to investigate the correlation of the various parameters of the RVRTC flow-volume curve with the other parameters of ILF testing, the parameter values were further transformed into Z-scores. Except for Z-FEV_0.5_/FVC, all RVRTC parameters moderately and positively correlated with Z-Vmax_FRC_, Z-Rrs, and Z-Crs (Table 3). Through the transformation into Z-scores, the parameters of the RVRTC flow-volume curves in particular displayed a relatively higher correlation with Vmax_FRC_ (all P < 0.001). The scatter plots and regression curves of the Z-score correlations are presented in Fig. 2.

**Table 3:** Correlations between various parameters of infant lung function.

*Pearson correlation*	Z-Rrs	Z-Crs	Z-Vmax_FRC_
Z-FVC	0.289**	0.440**	0.395**
Z-FEV_0.4_	0.418**	0.348**	0.384**
Z-FEV_0.5_	0.444**	0.368**	0.466**
Z-FEV_0.75_	0.434**	0.433**	0.484**
Z-FEV_1_	0.341**	0.432**	0.446**
Z-FEF_25–75_	0.429**	0.307**	0.429**
Z-FEF_75_	0.332**	0.410**	0.463**
Z-FEF_50_	0.383**	0.283*	0.426**
Z-FEV_0.5_/FVC	0.263**	—	—
Z-PEF	0.438**	0.225*	0.359**

*P < 0.05. **P < 0.01.

## Discussion

To the best of our knowledge, this is the first study to provide ILF testing reference data for Asian infants by using the full forced expiration manoeuvre. This longitudinal assessment was conducted in Taiwanese infants aged from 5 to 26 months. In the regression models for all RVRTC parameters, body length was found to be the most vital independent variable. Compared with the transformed Z-scores of ILF parameters, except for those measured while performing partial forced expiration (e.g., Vmax_FRC_), the RVRTC flow-volume curve lowly correlated with the parameters obtained from other ILF manoeuvres.

RVRTC technique is a revision of tidal RTC manoeuvres that passively inflated infant’s lung toward total lung capacity before applying RTC. The RVRTC technique enables a full forced expiration, and can yield a similar flow-volume curve as adult’s spirograms. Besides, RVRTC technique may have higher repeatability and reproducibility than tidal RTC technique. Previously, due to different setting of airway inflation pressure, the expiratory volume and flow varied a lot among laboratories. To facilitate collaboration and comparison of results between laboratories, ATS/ERS has recommended that the inflation pressure during RVRTC should be standardized to 30 cm H_2_O (2.94 kPa). Similar to the quality-control requirement of spirometry in preschool children, at least two technically acceptable flow-volume curves should be reproducible within 10% of the highest^5^.

**Figure 3:**
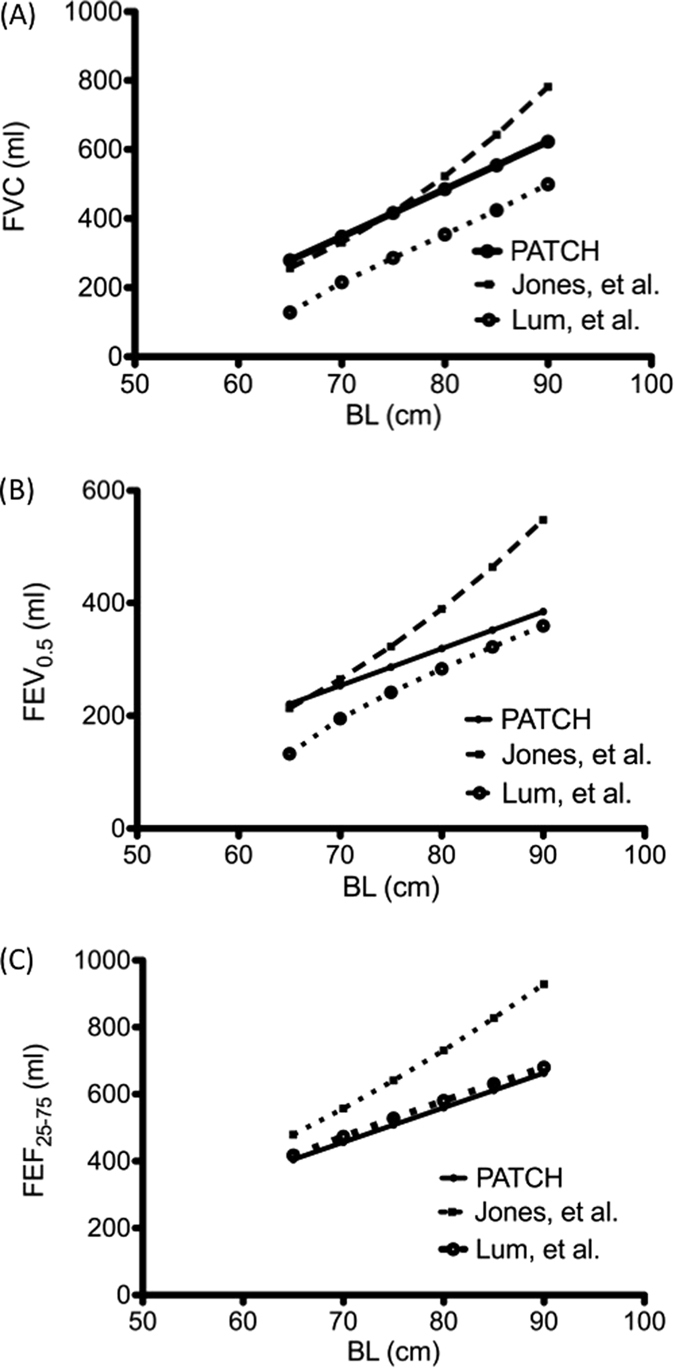
Comparison of various reference curves. Present regressions of forced vital capacity (FVC) (**A**), forced expiratory volume at 0.5 (FEV_0.5_) (**B**), and forced midexpiratory flow (FEV_25–75_) (**C**) in the study compared with previously published reference data.

Jones *et al*. developed the Caucasian reference of ILF by using RVRTC manoeuvres from ‘in-house’ equipment^8, 15^. However, after publication of the ATS/ERS guidelines^5, 8, 12^, commercial equipment was gradually applied in various Western centres. Currently, the Jaeger Masterscreen BabyBody is the only available commercial equipment and has been used worldwide for ILF assessment. Lum *et al*. have recently revealed significant differences between the references of ‘in-house’ and commercial equipment^8, 12, 16^. The use of unfitted reference data can result in the over- or underestimation of a predicted value depending on the equations used and disorientation of clinical judgment. As presented in Fig. 3, we compared the means of our reference data with those reported in the literature. Except for the predictive curve of FVC, the predictive values of FEV_0.5_ and FEF_25–75_ in our study approximated those reported by Lum *et al*., who used the same Jaeger’s machine used in our study. In addition, studies have claimed that reference equations for infant respiratory function are ethnicity-specific^11, 13^. Therefore, some differences were observed between the predictive values of FVC obtained in our study and those reported by Lum *et al*. (Fig. 3). The present study provides the first Jaeger-specific reference data for the infants of Chinese ethnicity.

Ethnic difference in lung function have been reported continually^17, 18^. The predicted values of FVC and FEV_1_ in Caucasians are larger than those of Asians and African-Americans. These differences are small in young age, but becomes significant bigger after young adult^18^. In adults, larger lung volumes and stronger respiratory muscle power in Caucasians, which affected by exercise, nutrition and overall health status, have been supposed to contribute to these differences^19^. However, in population of young children (less than 3 years of age), the growth patterns of both body weight and length are comparable between the Taiwanese and World Health Organization references^20^. It may hint that the proportion of body muscle mass all is similar in young children with various races. Therefore, although disparities exited between our and previous studies (Fig. 3), no evidence revealed that Asian infants have poor performance in FVC and FEV_0.5_ than Caucasian infants.

In adults and school-aged children, FEV_1_ is used as a vital parameter for assessing airway function. However, Bacharier *et al*. reported that FEV_1_ is very insensitive for classifying the severity of paediatric asthma, especially in young children^21^. Therefore, studies have advocated the use of FEV_0.75_, FEV_0.5_, and FEV_0.4_ to investigate airway function in young children and infants^22–24^. Although FEV_1_/FVC has been considered more sensitive for monitoring airflow limitation^15^, FEV_0.5_/FVC (or FEV_0.75_/FVC), instead of FEV_1_/FVC, appears to be a more suitable proxy index for detecting and monitoring airway function in very young children^25^. However, the value indicating airflow limitation depends highly on age when FEV_1_/FVC is used as an index. The normal value of this index for children is higher than that for adults^15^. Similarly, the results of our study as well as those of previous studies have shown that the value of FEV_0.5_/FVC is inversely related to body length^8, 12^. Therefore, further investigation is warranted to determine whether this test can be applied in general practice to identify young children with airflow limitation.

Through the use of spirometry, FEF_25–75_ is considered more sensitive than FEV_1_ for detecting small airway dysfunction^16^. In addition, studies have reported that FEF_25–75_ is a suitable proxy index for monitoring small airway function in children with asthma and smoke exposure^2–4, 26, 27^. Because the pathological lesions of respiratory diseases, such as bronchopulmonary dysplasia, are majorly located in the distal airway and alveoli^6, 7, 28^, our findings may offer a reliable local reference for future studies investigating young infants.

ILF testing has been widely used in research and clinical practice. Initially, tidal breathing analysis, respiratory system mechanics, forced expiration flow-volume curves, and lung volume measurements were used to evaluate respiratory function. Low T_pef_/T_e_ (<20%) in infancy has been reported to be related to the development of asthma in school age children^9, 29^. In our study, the infants with T_pef_/T_e_ < 20% (n = 24) had significantly lower Z-FEV_50_ than did those with T_pef_/T_e_ ≥ 20% (n = 83; P < 0.05). However, no significant association was observed between T_pef_/T_e_ and the other parameters of the RVRTC flow-volume curve.

The measurement of passive mechanics depends on the initiation of the Hering–Breuer reflex. Rrs and Crs can be analysed without any involvement of respiratory muscles. Although Harrison *et al*. has reported that Rrs in infancy is predictive of future FEV_1_ and FEF_25–75_ measured at the age of 6 years^10, 11, 30^, both Rrs and Crs only moderately correlated with most parameters of the RVRTC flow-volume curve in our study.

Vmax_FRC_, which is measured in tidal breathing analysis, is commonly used in clinical practice because of its easy applicability and accessibility. Although the relatively high variability of Vmax_FRC_ has been criticised^8, 12, 31^, our previous study has revealed that the upper and lower limits of agreement were 74% and 126%^1, 5^, respectively, suggesting that intraindividual variability was within the acceptable range. In the present study, we observed that the Vmax_FRC_ value was relatively higher correlated with the parameters of the RVRTC flow-volume curve, especially the values of FEV_0.75_ and FEV_0.5_. Therefore, Vmax_FRC_ remains a possible surrogate for labs in which RVRTC is unavailable.

Studies have indicated that maternal smoking might adversely affect the respiratory function of infants^1, 8, 32^. However, in our present study, only three mothers (3.1%) reported smoking during pregnancy. In addition, in this study as well as many previous studies^1, 5, 33–36^, various parameters have exhibited no significant differences between infants with and without smoke exposure. Moreover, in the present study, the history of smoke exposure was obtained through a self-report questionnaire instead of being quantitatively measured. Both the low number of infants with smoke exposure and the unreliable history of smoke exposure can hinder the analysis of the effects of smoke exposure.

This study has some potential weaknesses, which may limit its general application. First, our study group was recruited from a local community- and hospital-based practice and was not randomly sampled from the general population. Hence, population selection bias may be present because most enrollees live in Keelung City, which is located in Northern Taiwan. Nevertheless, our study group was similar to the normal local population in terms of various demographic characteristics (Table 1); this can largely increase the generalisability of these reference equations. Second, the number of participants with a body length of <65 cm was relatively low (only 4 infants). This would result in a broader range of upper and lower 95% confidence limits. Thus, the reference equations of this study should be cautiously applied in children with a body length of <65 cm. Furthermore, because of significant differences in the reference value among different studies using different equipment, our reference equations should be carefully used to interpret ILF data obtained using equipment other than Jager’s machine.

In conclusion, our study provides reference data and equations for the measurement of full forced expiration using RVRTC manoeuvres in Taiwanese infants of Chinese ethnicity. This reference is also the first equipment-specific RVRTC prediction equations for an Asian population. Our results revealed that the parameters of RVRTC manoeuvres correlate moderately with the parameters of other ILF manoeuvres. Our race- and equipment-specific data may improve the diagnosis and monitoring of respiratory diseases in infants of Chinese ethnicity.

## Methods

### Study population and data collection

Records were obtained from an ongoing prospective birth cohort study called the Prediction of Allergies in Taiwanese Children (PATCH) from January 2013 to December 2015. PATCH is an unselected, population-based birth cohort study investigating risk factors for immune-related and allergic diseases in children in Keelung City (Northern Taiwan). Detailed descriptions of the recruitment process and data collection have been reported previously^1, 21^. Neonates born prematurely (gestational age of <36 weeks), those with major birth defects or congenital structural anomalies of the upper airway, those who were haemodynamically unstable, and those with a history of severe lower airway infection with intensive care unit admission were excluded from the study. The study was approved by the Institutional Review Board of Chang Gung Memorial Hospital (IRB reference number 100-0286B). Our study was performed in line with the Declaration of Helsinki and International Committee of Harmonisation good clinical practice. All the written informed consents were provided from the parents or legal guardians of neonates.

### ILF testing

Measurements were performed in healthy infants without a respiratory tract infection for at least 3 weeks. Before the tests, their body weight was measured and crown–heel length was obtained on a measuring board. Subsequently, infants were sedated with oral chloral hydrate (50–75 mg/kg) and placed in the supine position, with the neck mildly extended. ILF testing was performed using the Jaeger Masterscreen BabyBody Paediatrics System (CareFusion, Hoechberg, German). The equipment conforms to the ATS/ERS recommendations^5, 22–24, 33–36^.

### Tidal breathing analysis, lung mechanics, and tidal forced expiration

Detailed procedures and data collection methods involved in tidal breathing analysis, respiratory mechanics, and forced tidal expiration have been provided previously^1, 15^. The ratio of time to peak expiratory flow to total expiratory time (T_pef_/T_e_) in the tidal breathing analysis, the resistance and compliance of the respiratory system (Rrs and Crs, respectively) in respiratory mechanics, and Vmax_FRC_ in forced tidal expiration were collected for subsequent analysis.

### Raised-volume forced expiration

The RVRTC technique was performed in accordance with the ATS/ERS guidelines^5, 25^. The jacket inflation pressure was set to match the value of the previous forced tidal expiration. Forced expiratory flows were initiated from a lung volume at which the airway pressure was 30 cm H_2_O (V30) and proceeded to the residual volume. Inflating the respiratory system to V30 three to five times prior to the forced expiratory manoeuvre inhibited the respiratory effort during forced expiration. Occlusion of the expiratory valve resulted in the inflation of the respiratory system to an airway pressure of 30 cm H_2_O. Forced expiration was initiated at this elevated lung volume by rapidly inflating the jacket, which was wrapped around the chest and abdomen of the infants. At least two technically satisfactory and reproducible (within 10%) maximal RVRTC expiratory flow-volume curves were obtained. The following measurements were performed: forced vital capacity (FVC); forced expiratory volume at 1, 0.75, 0.5, 0.4, and 0.3 seconds (FEV_1_, FEV_0.75_, FEV_0.5_, FEV_0.4_, and FEV_0.3_, respectively); forced midexpiratory flow (FEF_25–75_); forced expiratory flow at exhaled 75% and 50% of vital capacity (FEF_75_ and FEF_50_, respectively); and peak expiratory flow (PEF).

### Statistical Analysis

Normal probability plots were used to evaluate the normality assumption of the lung function parameters. All parameters followed an acceptably normal distribution; therefore, parametric analyses were performed. Spearman correlation analysis was used to delineate the relationship between the lung function parameters and the demographic data of participants. Stepwise multiple regression was used to determine the characteristics that significantly improved the fraction of observed differences in the dependent variables. Subsequently, prediction equations were developed. Simple linear regressions were performed to investigate the correlation between the Z-scores of the various parameters of lung function testing. Results with *P* < 0.05 were considered statistically significant. All statistical analyses were performed using IBM SPSS Statistics, Version 20 (Armonk, NY, USA).
